# Hybrid self-optimized clustering model based on citation links and textual features to detect research topics

**DOI:** 10.1371/journal.pone.0187164

**Published:** 2017-10-27

**Authors:** Dejian Yu, Wanru Wang, Shuai Zhang, Wenyu Zhang, Rongyu Liu

**Affiliations:** School of Information, Zhejiang University of Finance and Economics, Hangzhou, Zhejiang, China; Jiangnan University, CHINA

## Abstract

The challenge of detecting research topics in a specific research field has attracted attention from researchers in the bibliometrics community. In this study, to solve two problems of clustering papers, i.e., the influence of different distributions of citation links and involved textual features on similarity computation, the authors propose a hybrid self-optimized clustering model to detect research topics by extending the hybrid clustering model to identify “core documents”. First, the Amsler network, consisting of bibliographic coupling and co-citation links, is created to calculate the citation-based similarity based on the cosine angle of papers. Second, the cosine similarity is also used to compute the text-based similarity, which consists of the textual statistical and topological features. Then, the cosine angle of the linear combination of citation- and text-based similarity is considered as the hybrid similarity. Finally, the Louvain method is applied to cluster papers, and the terms based on term frequency are used to label clusters. To test the performance of the proposed model, a dataset related to the data envelopment analysis field is used for comparison and analysis of clustering results. Based on the benchmark built, different clustering methods with different citation links or textual features are compared according to evaluation measures. The results show that the proposed model can obtain reasonable and effective clustering results, and the research topics of data envelopment analysis field are also analyzed based on the proposed model. As different features are considered in the proposed model compared with previous hybrid clustering models, the proposed clustering model can provide inspiration for further studies on topic identification by other researchers.

## Introduction

Clustering or mapping of scientific papers is an important area in scientometric research [1]. Clustering analysis is useful for detecting research topics and revealing scientific structure and dynamics, which can aid in systematic understanding of research fields. Bibliometric methods have been used to analyze research topics and scientific structure in different research fields [2–5]. There are also some studies combining bibliometric methods with clustering methods for clustering papers based on citation links, textual approach, and a combination of citation links and textual approach. These are known as citation-based method, text-based method, and hybrid clustering, respectively. Some related works are listed in Table 1 and are discussed in detail here.

**Table 1:** Related work of clustering methods based on citation links and textual features.

Methods	Similarity computation	References
**Citation-based methods**	Co-citation network	Small [7]
	Bibliographic coupling network	Kessler [8]
	Amsler network	Amsler [9]
**Text-based methods**	Co-word analysis	Callon et al. [11]; Radhakrishnan et al. [12]; Yu et al. [13]
	Text mining methods	Boyack et al. [14]
**Hybrid clustering methods**	Combination of co-citation and co-word analysis	Braam et al. [17,18]
	Combination of bibliographic coupling and TF-IDF	Glänzel and Thijs [10,19]; Liu [21]
	Combination of cross-citation network and TF-IDF	Liu et al. [16]; Meng et al. [20]

The main citation-based methods are direct citation, co-citation, bibliographic coupling, and Amsler. According to Janssens et al. [6] and Liu et al. [1], the most widely used method is co-citation, which was proposed by Small [7], although bibliographic coupling was discovered earlier by Kessler [8]. Co-citation can be considered when there are common in-links between two papers, and bibliographic coupling can be considered when there are common out-links between two papers. Amsler [9] proposed a combination of bibliographic coupling and co-citation that considers both the common out- and in-links between two papers. The main advantage of the citation-based methods is their discrimination power; however, these methods tend to “underestimate” the relationship between papers [10].

A popular text-based method is co-word analysis [11], which analyzes the occurrence of words extracted from papers in an indexing database based on text mining techniques. Previous studies applied co-word analysis to cluster and analyze research topics in different scientific fields [12,13]. There are additional text mining methods that are applied to identify important terms in a paper in bibliometric research [14], such as term frequency, inverse document frequency, and term frequency-inverse document frequency (TF-IDF), which are popular text mining methods based on term frequency. The analysis of terms or keywords in two papers can be used not only to calculate the text-based similarity, but also to label the research topic of the paper. However, only term frequency is considered as the importance of terms is unilateral, and some high-frequency terms cannot represent the specific research fields [15].

Both citation- and text-based methods have their respective advantages and disadvantages. Citation-based methods can reveal the link structure and link relationship between papers but neglect the text feature between papers, whereas text-based methods only consider the text feature between papers, but neglect the links between papers. Hybrid clustering methods, which combine citation- and text-based methods have been studied to improve the performance of scientific mapping and clustering [1,16].

Braam et al. [17,18] first proposed the hybrid clustering of co-citation and co-word analysis for scientific mapping from the perspective of structural aspects and dynamical aspects, respectively. Afterward, there are an increasing number of interesting studies regarding the combination of citation- and text-based methods for clustering in different ways. Glänzel and Thijs [10,19] used the cosine measure to calculate both the citation- and text-based similarities based on bibliographic coupling and TF-IDF, respectively, and then combined the cosine angles underlying the citation- and text-based similarities to detect “core documents.” Liu et al. [16] used a cross-citation network to construct a link structure, where the text-based similarity, calculated as the cosine angle of the documents based on the TF-IDF weight of terms, was considered as the edge strength in a coupled graph; then, the link structure was coupled with the edge strength to cluster papers using the Louvain method. Meng et al. [20] proposed a multi-view clustering method, which uses a cosine similarity based on the TF-IDF of the terms as the text-based similarity, and adopted a cross-citation relation between papers to compute the citation-based similarity. Then, a simple linear combination of these two similarities was used as the integrated similarity to cluster journals. Liu [21] combined the bibliographic coupling with the context information of the references to compute the similarity between papers in the biomedical field. Silva et al. [22] constructed a citation network for clustering, extracted keywords to define the cluster topics, and combined these with network topological metrics to analyze the relatedness between topics.

Previous studies on hybrid clustering methods have contributed to improving the performance of clustering. Many researchers tend to use one type of citation link to compute the citation-based similarity and a text statistical feature to compute the text-based similarity. Then, a simple linear combination of these two similarities between papers is represented as the integrated similarity to cluster documents. However, there are three issues to be noted. First, Calado et al. [23] and Couto et al. [24] found that different distributions of citation links in direct citation, co-citation, and bibliographic coupling networks can influence the clustering performance. Second, previous studies usually only took textual statistical features based on TF-IDF as the text-based similarity, whereas Chen and Xiao [15] pointed out that only taking the term frequency as the importance of terms is not sufficient. Third, the simple linear combination of hybrid similarity might neglect different distributional characteristics of different datasets [25].

To solve these three issues, a hybrid self-optimized clustering model based on citation links and textual features is proposed in this study by extending the hybrid clustering model of Glänzel and Thijs [10]. First, the Amsler network that considers both the common out- and in-links between papers is created to address the preceding one, and then it is used to calculate the citation-based similarity by extending the measurement of the bibliographic coupling strength by Glänzel and Czerwon [26] based on the angle between vectors. Second, to solve the second issue, the statistical feature based on TF-IDF and the topological feature based on accessibility of terms extracted from the title and abstract of papers are combined to calculate the text-based similarity using the cosine measurement based on the research by Amancio [27], which showed that adding a textual topological property can improve the performance of conventional textual statistical methods. Third, to solve the third issue, Glänzel and Thijs [10] proposed a linear combination of the angles of similarities. Following the proposal of this hybrid method, some studies conducted further analysis and proposed applications [19, 28–30]. In this study, we also use the cosine angles underlying the linear combination of the citation- and text-based similarities, which are determined to represent the hybrid similarity between papers. Finally, the clustering result is obtained by applying the Louvain method [31], and the terms based on term frequency are used to label the research topics of the obtained clusters.

In addition, there are also other effective clustering methods that have been proposed and applied to text clustering, such as the soft subspace clustering (SSC) method, which has been extended and evaluated in text clustering. Jing et al. [32] used the weights calculated by the extended *k*-means clustering methods automatically to detect subsets of important dimensions. Deng et al. [33] proposed a new enhanced SSC method using within-class compactness and between-cluster separation based on a developed optimization objective function. Wang et al. [34] proposed a novel extended SSC algorithm, which introduced a partition index into the objective function and combined the concepts of hard and fuzzy clustering. These studies all applied the extended SSC methods to cluster textual data and evaluated their effective performance. However, we chose the Louvain method to cluster papers because it does not require the number of clusters to be set beforehand as it is an efficient and self-optimization clustering method based on modularity [16]. The detailed reasons are introduced in next section.

The remainder of this paper is organized as follows. We first illustrate the details of the proposed model. Then we present the dataset related to the data envelopment analysis (DEA) field and the experimental results. Finally, we conclude the paper and discuss future works.

## Model and methodology

First, to facilitate understanding of the proposed model, the symbols used in this study and their corresponding explanations are listed in Table 2.

**Table 2:** Symbols used in this study and their corresponding explanations.

Symbols	Explanations
*α*	Weight of citation-based similarity
*β*	Weight of text-based similarity
*λ*	Weight of statistical feature of terms
*p*_*i*_, *p*_*j*_	Two papers in the example
**A**	The matrix of the total links and their strength of all pairs of papers based on the Amsler network
**L**	Matrix of the citation-based similarity between papers
$f_{t_{i},p}$	Frequency of term *t*_*i*_ in paper *p*
$LS_{\left(p_{i},p_{j}\right)}$	Citation-based similarity between *p*_*i*_ and *p*_*j*_.
*N*_*p*_	Total number of papers
$df_{t_{i}}$	Number of papers containing term *t*_*i*_
*t*_*i*_, *t*_*j*_	Two nodes of the terms connected in paper *p*
$TF\text{-}IDF_{\left(t_{i},p\right)}$	Statistical feature of term *t*_*i*_ in paper *p*
$I_{\left(t_{i},p\right)}$	Importance of terms
*M*	Total number of performed walks
$W_{\left(t_{i}\to t_{j}\right)}$	Number of times that node *t*_*i*_ reaches node *t*_*j*_ after *h* steps
$P_{h}{}_{\left(t_{j},t_{i}\right)}$	Transition probability that node *t*_*i*_ reaches node *t*_*j*_ after *h* steps of a self-avoiding walk
*E*_*h*_(Ω,*t*_*i*_)	Diversity of node *t*_*i*_ after *h* steps
$OA_{h}{}_{\left(t_{i},p\right)}$	Outward accessibility of node *t*_*i*_ after *h* steps
$TS_{\left(p_{i},p_{j}\right)}$	Text-based similarity between papers *p*_*i*_ and *p*_*j*_
*k*	Total number of terms appearing in papers *p*_*i*_ and *p*_*j*_.
$ES_{\left(p_{i},p_{j}\right)}$	Hybrid similarity between *p*_*i*_ and *p*_*j*_
$E_{p_{i}p_{j}}$	Weight of the edges between vertex *p*_*i*_ and vertex *p*_*j*_
$w_{p_{i}}$, $w_{p_{j}}$	Sum of the weights of the edges attached to vertexes *p*_*i*_ and *p*_*j*_, respectively
$c_{p_{i}}$, $c_{p_{j}}$	The clusters to which vertexes *p*_*i*_ and *p*_*j*_ belong to, respectively
$w_{p_{i},in}$	Sum of the weights of the links from *p*_*i*_ to nodes on a community *C*
*m*	Sum of the weights of all the links in the network
Σ_in_	Sum of the weights of the links inside a community *C*
Σ_tot_	Sum of the weights of the links incident to nodes in a community *C*

Based on the previous study by Glänzel and Thijs [10], some improvements to the hybrid clustering method are made in this current study. On one hand, the Amsler network of papers is constructed to calculate the citation-based similarity, which means that it is calculated by considering both the common out- and in-links between papers, while only the bibliographic coupling strength is used to compute the citation-based similarity by Glänzel and Thijs [10]. On the other hand, the calculation of the text-based similarity considers not only the frequency of terms extracted from the paper titles and abstracts, but also the topological characterization of the term adjacency network, whereas only the textual frequency feature is considered in the textual similarity by Glänzel and Thijs [10]. Finally, the Louvain method [31] is applied to cluster papers based on the hybrid similarity between papers.

**Fig 1:**
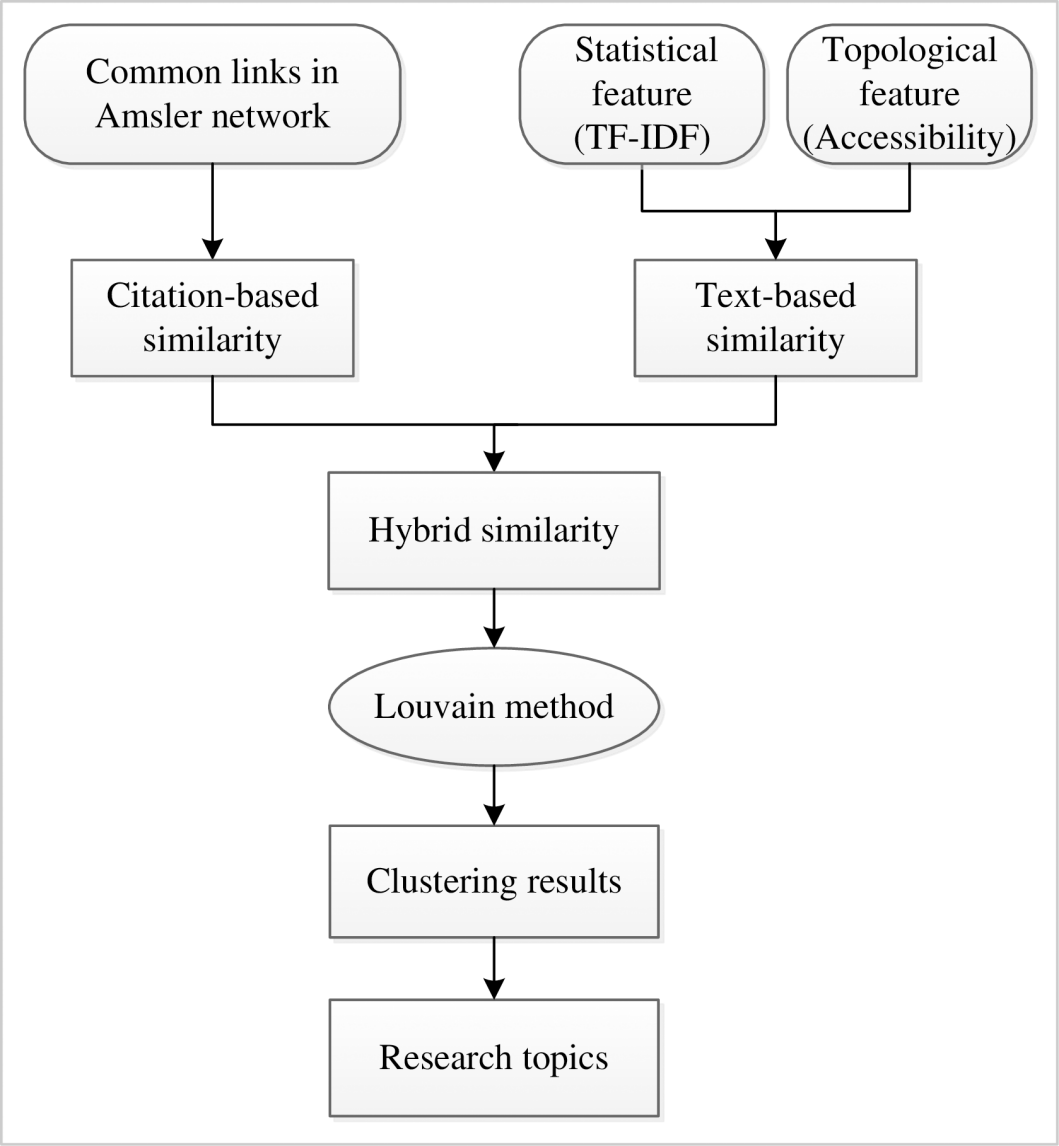
Flowchart of the proposed hybrid self-optimized clustering model.

The advantages of the proposed model are reflected in three main aspects: (a) it can make citation-based and text-based methods complementary to improve the efficiency and overcome some limitations of these two methods [10]; (b) considering both the common out- and in-links between papers can express the bibliometric relations between papers more accurately [24]; and (c) adding the topological feature into the statistical feature of terms can improve the performance of existing textual statistical methods [27]. Moreover, the Louvain method is an efficient and self-optimal clustering method based on modularity; thus, it does not require the number of clusters to be set beforehand. Once the clustering result is obtained, the clusters can be labeled according to the terms extracted from the title and abstract based on the term frequency. The proposed model is illustrated in Fig 1 and the detailed methods are introduced below.

### Citation-based analysis

**Fig 2:**
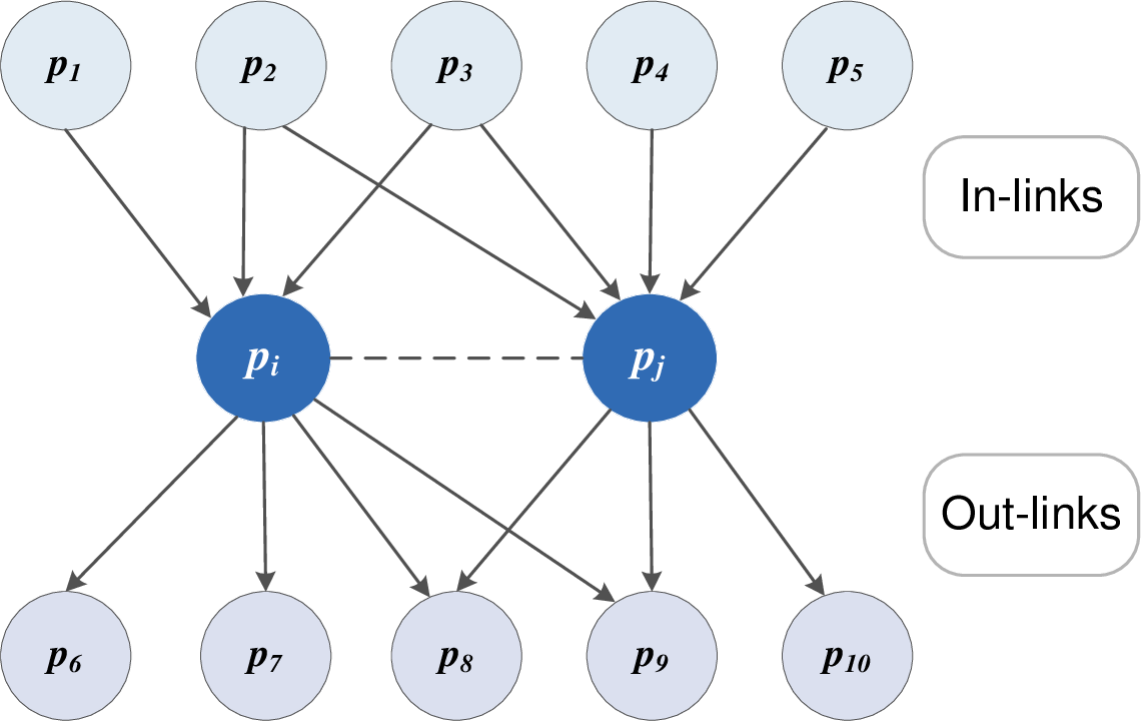
In-links and out-links between two papers. *p*_*1*_, *p*_*2*_, and *p*_*3*_ represents the citations of the paper *p*_*i*_, while *p*_*2*_, *p*_*3*_, *p*_*4*_, and *p*_*5*_ represent the citations of the paper *p*_*j*_; therefore, *p*_*2*_ and *p*_*3*_ are the common in-links between *p*_*i*_ and *p*_*j*_. Similarly, *p*_*6*_, *p*_*7*_, *p*_*8*_, and *p*_*9*_ represent the references of the paper *p*_*i*_, while *p*_*8*_, *p*_*9*_, and *p*_*10*_ represent the references of the paper *p*_*j*_; therefore, *p*_*8*_ and *p*_*9*_ are the common out-links between *p*_*i*_ and *p*_*j*_.

First, to obtain the citation-based similarity between two papers, the Amsler network was constructed based on Amsler’s method [9]. According to Amsler [9] and Calado et al. [23], co-citation and bibliographic coupling can be combined to measure the similarity, which means that two papers *p*_*i*_ and *p*_*j*_ are related if any of the three following conditions is met: (i) *p*_*i*_ and *p*_*j*_ are co-cited by the same paper, (ii) *p*_*i*_ and *p*_*j*_ cite the same paper, and (iii) *p*_*i*_ cites a third paper that cites *p*_*j*_. According to the definitions of co-citation [7] and bibliographic coupling [8], the relationship of co-citation between two papers can be used to measure the number of common in-links of two papers, whereas bibliographic coupling can be used to measure the number of common out-links of two papers. It should be noted that only the first two conditions in the Amsler network are considered in this study. Moreover, the common out- and in-links between two papers depend on the common citations and references, respectively. An example is shown in Fig 2.

Based on the method introduced by Glänzel and Czerwon [26] whereby the citation-based similarity was calculated using the cosine angles between vectors based on bibliographic coupling, a matrix **A** representing the total links and their strength of all pairs of papers was built based on the Amsler network. The diagonal elements of matrix **A** represent the total number of links of each paper, i.e., the number of references and citations, and the corresponding elements located in the *i*th row and *j*th column represent the number of common out- and in-links between *p*_*i*_ and *p*_*j*_. Then, another matrix, **L**, representing the citation-based similarity between papers can be created as Eq (1), according to Glänzel and Czerwon [26]:

$$L=[LS_{\left(p_{i}p_{j}\right)}]=\mathrm{Diag}{\left(A\right)}^{-1/2}\cdot A\cdot \mathrm{Diag}{\left(A\right)}^{-1/2}.$$ (1)

According to Glänzel and Czerwon [26], matrix **L** contains Salton’s cosine measure of the link strength of all pairs of papers. The difference between this study and theirs is that the link strength is calculated based on the Amsler network rather than on the bibliographic coupling network. Although Glänzel and Czerwon [26] showed that the bibliographic coupling network has several advantages compared to the co-citation network, some studies [23,24] also verified that different distributions of citation links have different influences on the citation-based clustering. Therefore, to weaken the influence of the different distributions of citation links, both the common out- and in-links between pairs of papers are involved in the computation of citation-based similarity in this study.

### Text-based analysis

According to Amancio [27], recently, complex network methods have proved useful to create several language models. Some complex network methods were devised to improve the performance of statistical methods. The results of several cases indicated that the hybrid methods of statistical features and topological properties outperformed the results when only the statistical or network methods were used. Inspired by Amancio [27], the statistical feature of terms is combined with the topological feature of term adjacency network as the importance of terms. Then, the text-based similarity between two papers can be calculated using Salton’s cosine measure [35].

#### Statistical feature of terms

The TF-IDF method [36] is applied to compute the statistical weight of terms. Using the TF-IDF can identify important terms based on term frequency and inverse document frequency. The statistical feature $TF\text{-}IDF_{\left(t_{i},p\right)}$ of term *t*_*i*_ in a paper *p* can be calculated using the TF-IDF method as:

$$TF\text{-}IDF_{\left(t_{i},p\right)}=f_{t_{i},p}\times \log\frac{N_{p}}{df_{t_{i}}}.$$ (2)

Considering that the TF-IDF method only takes into account the frequency of terms and ignores the influence of the terms’ interconnectivity in the paper, the authors added the topological features of terms based on the term adjacency network, as shown in the next sub-section.

#### Topological feature of terms

According to Newman [37], there are several popular topological metrics in a network, such as degree, betweenness, closeness, average shortest path length, and accessibility. Based on the good performance of the accessibility metric in previous studies [27,38,39], the accessibility metric [40] is applied to analyze the topological feature based on the term adjacency network in this study, which considers both the topology and dynamics of the network. According to Travençolo and Costa [40], accessibility is the normalization of diversity entropy, which can be used to evaluate the relative accessed frequency of one specific node. Furthermore, it can be divided into outward accessibility and inward accessibility, in which the former quantifies the diversity with which a node accesses the other nodes, and the latter quantifies the frequency at which the other nodes access the specific node. Considering that the accessibility applied in this study is aimed at representing the topological feature rather than the frequency of terms, the outward accessibility is chosen for further computation. To define this metric, the following definitions are considered [40].

Let *G*^*T*^ be an unweighted and undirected term adjacency network with *N*_*t*_ nodes based on the terms extracted from a paper *p*. The nodes in the network represent the terms, and the edge between two nodes *t*_*i*_ and *t*_*j*_ represents two nodes connected in the paper *p*, i.e., the two nodes are adjacent. $P_{h}{}_{\left(t_{j},t_{i}\right)}$ is the transition probability that node *t*_*i*_ reaches node *t*_*j*_ after *h* steps of a self-avoiding walk, which is calculated as:

$$P_{h}{}_{\left(t_{j},t_{i}\right)}=\frac{W_{\left(t_{i}\to t_{j}\right)}}{M}.$$ (3)

According to Amancio [27], the standard deviation of the accessibility at the third level results in the best topological feature; therefore, *h* is also set as 3 in this study.

Then, the diversity *E*_*h*_(Ω,*t*_*i*_) of node *t*_*i*_ after *h* steps is calculated as:

$$E_{h}(\Omega ,\;t_{i})=-\sum_{t_{j}=1}^{N_{t}}\left\{ \begin{array}{l} \;0\;\mathrm{if}\;P_{h}{}_{\left(t_{j},t_{i}\right)}=0,\\ \;P_{h}{}_{\left(t_{j},t_{i}\right)}\log P_{h}{}_{\left(t_{j},t_{i}\right)}\;\mathrm{if}\;P_{h}{}_{\left(t_{j},t_{i}\right)}\neq 0. \end{array}\right.$$ (4)

Finally, the outward accessibility $OA_{h}{}_{\left(t_{i},p\right)}$ of node *t*_*i*_ after *h* steps is calculated as:

$$OA_{h}{}_{\left(t_{i},p\right)}=\frac{\exp(E_{h}(\Omega ,t_{i}))}{N_{t}-1}.$$ (5)

#### Text-based similarity computation

Once the statistical and topological feature of terms in the term adjacency network have been obtained, the importance of terms can be calculated as:

$$I_{\left(t_{i},p\right)}=\lambda \cdot TF\text{-}IDF_{\left(t_{i},p\right)}+(1-\lambda )\cdot OA_{h}{}_{\left(t_{i},p\right)},$$ (6)

where *λ* ϵ [0,1]. The value of this parameter is explained later in detail.

Then the text-based similarity $TS_{\left(p_{i},p_{j}\right)}$ between papers *p*_*i*_ and *p*_*j*_ can be calculated as the cosine of the angle between the term vectors by using the cosine measure [35], as shown below.

$$TS_{\left(p_{i},p_{j}\right)}=\frac{\sum_{t=1}^{k}I_{\left(t,p_{i}\right)}\times I_{\left(t,p_{j}\right)}}{\sqrt{\sum_{t=1}^{k}(I_{\left(t,p_{i}\right)}{)}^{2}}\times \sqrt{\sum_{t=1}^{k}(I_{\left(t,p_{j}\right)}{)}^{2}}}.$$ (7)

### Hybrid clustering using the Louvain method

#### Hybrid similarity

Inspired by the definition of hybrid similarity by Glänzel and Thijs [10], the hybrid similarity is also represented by the cosine angle of the linear combination of the aforementioned computation of citation- and text-based similarity. Therefore, the hybrid similarity between *p*_*i*_ and *p*_*j*_ ($ES_{\left(p_{i},p_{j}\right)}$) in this study can be calculated by the following formula:

$$ES_{\left(p_{i},p_{j}\right)}=\cos\;(\alpha \cdot \arccos\;(LS_{\left(p_{i},p_{j}\right)})+\beta \cdot \arccos\;(TS_{\left(p_{i},p_{j}\right)})),$$ (8)

where *α* ϵ [0,1], *β* ϵ [0,1], and *α* + *β* = 1.

#### Louvain method

Once the hybrid similarity between papers is obtained, the Louvain method can be applied to cluster papers based on the optimal modularity. The Louvain method [31] is a popular community detection method with excellent accuracy and rapidity based on modularity optimization. Modularity is usually used to detect the community structure for optimization methods in networks [41]. In this study, the hybrid similarities between papers are considered as the weight of the edges in the Amsler network, thus, the modularity (*Q*) is defined as [42]:

$$Q=\frac{1}{2m}\sum_{p_{i}p_{j}}\left[E_{p_{i}p_{j}}-\frac{w_{p_{i}}w_{p_{j}}}{2m}\right]\;\delta (c_{p_{i}}c_{p_{j}}),$$ (9)

$$w_{p_{i}}=\sum {}_{p_{j}}E_{p_{i}p_{j}},$$ (10)

where the δ function δ(*u*, *v*) is 1 if *u* = *v* and 0 otherwise and $m=\frac{1}{2}\sum {}_{p_{i}p_{j}}E_{p_{i}p_{j}}$.

Based on the modularity optimization, the clustering process of the Louvain method consists of two phases repeated iteratively [16,31]. First, each node is assigned to a different community, and then each node is moved to another community *C* based on the gained modularity. The second phase is repeated iteratively for all nodes on the network until the optimal assignment is achieved. During the first phase, the gain of modularity of one node moved from its community to another community is evaluated. The node is then placed in the community where its gained modularity is maximum, but only if this gain is positive. If there is no positive gain, the node will stay in its original community [31].

According to Blondel et al. [31], part of the algorithm’s efficiency results from the fact that the gain in modularity Δ*Q* is obtained by moving an isolated node into a community *C*, which can be computed by

$$\Delta Q=\left[\frac{\sum {}_{in}+2w_{p_{i},in}}{2m}-{\left(\frac{\sum {{}_{t}}_{ot}+w_{p_{i}}}{2m}\right)}^{2}\right]-\left[\frac{\sum {}_{in}}{2m}-{\left(\frac{\sum {{}_{t}}_{ot}}{2m}\right)}^{2}-{\left(\frac{w_{p_{i}}}{2m}\right)}^{2}\right].$$ (11)

There are several reasons why the Louvain method is chosen to cluster papers in this study. First, its excellent self-optimizing procedure based on modularity [42] means that it does not require the number of clusters to be set before the clustering process is conducted. Second, the Louvain method can take each paper as a vertex so that the clustering analysis can be implemented directly [16]. Third, Colliander and Ahlgren [43] and Meyer-Brötz et al. [30] found that it is easy to obtain considerably more similar distributions of clustering sizes when the Louvain method is applied to cluster documents with different parameter setting of similarities. Moreover, some existing studies have shown the efficient performance of the Louvain method, such as those by Liu et al. [16] and Meng et al. [20]. Based on the proposed hybrid clustering model, a case study related to the DEA field is discussed in the following section.

## Dataset and experimental results

### Dataset

In this study, we used the proposed model for clustering papers in the DEA field to detect its research topics. The dataset contains 7308 papers related to the DEA field, which was downloaded from the ISI Web of Science database on December 31, 2016. The retrieval method was employed by typing “data envelopment analysis” as the “Subject,” coupled with “Science Citation Index Expanded, SCIE,” and “Social Science Citation Index, SSCI” in the “More settings” option, with no time limitation. After the page jumped to the search results page, only the records of types “article” and “review” were retained, eventually leaving 7308 papers for use in this study. During the creation process of the Amsler network based on the common out-links and in-links between papers, five isolated nodes with no out-links and in-links were deleted from the dataset; therefore, the final dataset comprises 7303 papers.

**Fig 3:**
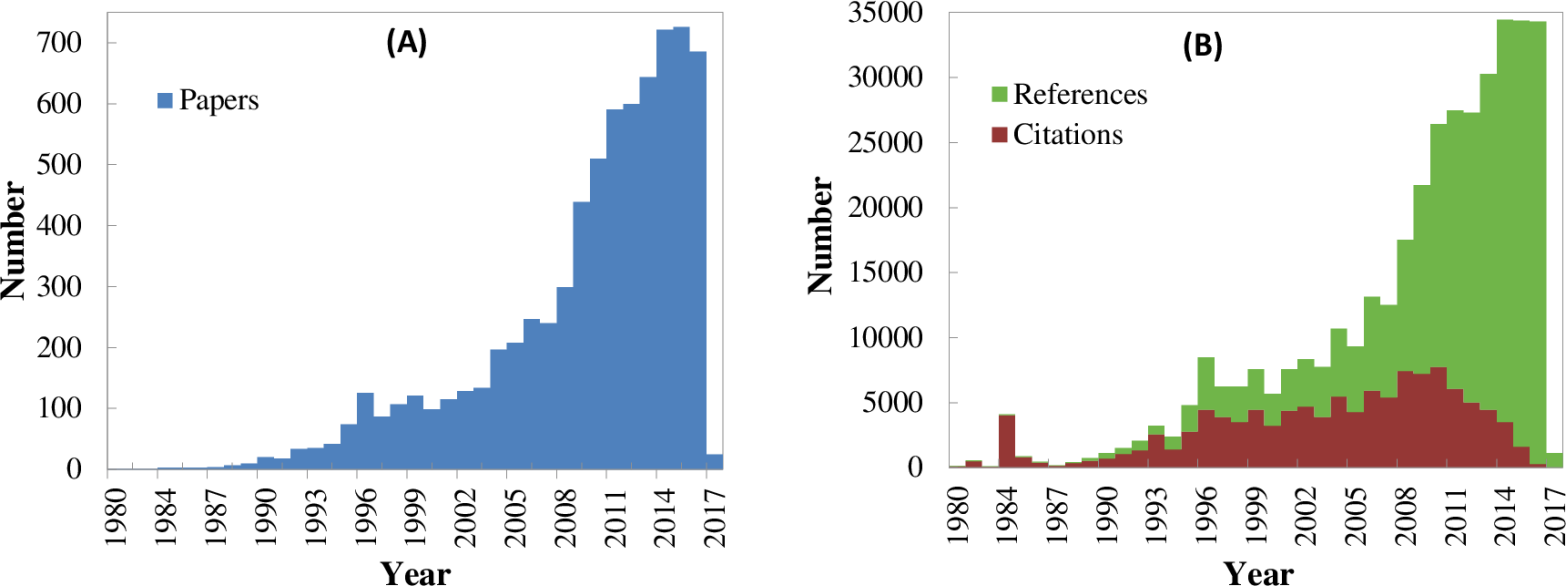
Basic statistics of DEA-related papers per year from 1980 to 2017. (A) Number of papers. (B) Number of citations and references of the papers.

Because the number of common out-links and in-links between papers is based on the citation links, i.e. the references and citations of papers, we generate the basic statistics of the papers. The number of papers per year from 1980 to 2017 is shown in Fig 3A, and the number of citations and references of the 7303 papers is shown in Fig 3B. It can be observed from Fig 3B that the number of references significantly increased in 2009, and the difference between the number of citations and references has continued increasing, particularly after 2009.

In addition, the pre-processing of text data was implemented using the “SnowballC” and “tm” packages in R project. First, the corpus of documents was constructed by extracting terms from the titles and abstracts of the 7303 papers. Then, the numbers, punctuation, whitespace, and stopwords were removed. Next, each remaining term was lemmatized using Porter's stemming algorithm. Finally, the pre-processed terms were obtained for further analysis.

### Benchmark and evaluation measures

Comparing different models with different parameter settings requires a benchmark, which represents the gold standard of the clustering results. Considering that the dataset used in this study does not have a standard clustering result, the method of building a benchmark needs to be given first. According to previous studies, there are several methods for building benchmarks. For instance, Ahlgren and Colliander [44] asked some experts to perform a subjective classification of 43 papers using the abstracts and titles extracted from the papers as the ground truth classification. Chen and Xiao [15] also asked several experts to detect a keyword dataset as a reference to quantitatively evaluate which method more closely approximates the experts’ selections. Couto et al. [24] used a text-based classification of each classification method as the benchmark; these are the k-nearest neighbor classifier using the cosine similarity, and the support vector machine classifier with TF-IDF. Considering that the expert-based baseline is time- and labor-consuming, and also has a certain degree of subjectivity, the authors used the benchmark built by Couto et al. [24] as a reference. Then, text-based clustering with cosine measure was conducted based on TF-IDF using the Louvain method, i.e., *α* = 0, *β* = 1, *λ* = 1 in the proposed model are set as benchmark for further analysis in this study.

The main evaluation measures applied in this study are the F1 measure, and rand index (RI). F1 measure is the harmonic mean between precision and recall, and is also widely used to measure clustering [45,46], which is calculated in the standard approach, i.e., Eq (12). Precision and recall are widely used in classification and clustering tasks for measuring the relevance [47], and are defined as Eqs (13) and (14) respectively. RI is used for measuring the agreement and disagreement between object pairs in different clusters, and is generally calculated using Eq (15) [48]. All these indices are used to measure the degree of matching between different clustering results and the benchmark.

$$F1=\frac{2\cdot precision\cdot recall}{precision+recall},$$ (12)

$$precision=\frac{tp}{tp+fp},$$ (13)

$$recall=\frac{tp}{tp+fn},$$ (14)

$$RI=\frac{tp+tn}{tp+fp+fn+tn},$$ (15)

where *tp* represents the number of pairs in the same cluster in the benchmark that is also clustered in the same cluster in the evaluated models, *fp* represents the number of pairs in different clusters in the benchmark that is clustered in the same cluster in the evaluated models, *tn* represents the number of pairs in different clusters in the benchmark that is also clustered in different clusters in the evaluated models, and *fn* represents the number of pairs in different clusters in the benchmark that is clustered in the same cluster in the evaluated models.

### Values of parameters

There are three parameters (*λ*, *α*, and *β*) that need to be further analyzed, as stated previously. *λ* is the weight of statistical feature of terms, which has an effect on the text-based similarity. Amancio [27] applied optimization heuristics to determine suitable values of the parameter, and found that *λ* = 0.15 can obtain a considerably reasonable classification and considered the topological feature of terms as the main feature. Therefore, the authors also set the value of *λ* as 0.15 based on the research result of Amancio [27]. Regarding the remaining parameters, *α* and *β*, their optimal values were obtained by using an iterative computation according to the values of evaluation measures of the clustering results. The values of *α* are set in the range of 0 to 1 with 0.05 as the interval used to analyze the distribution of the F1 measure and RI values of different clustering results based on the benchmark.

**Fig 4:**
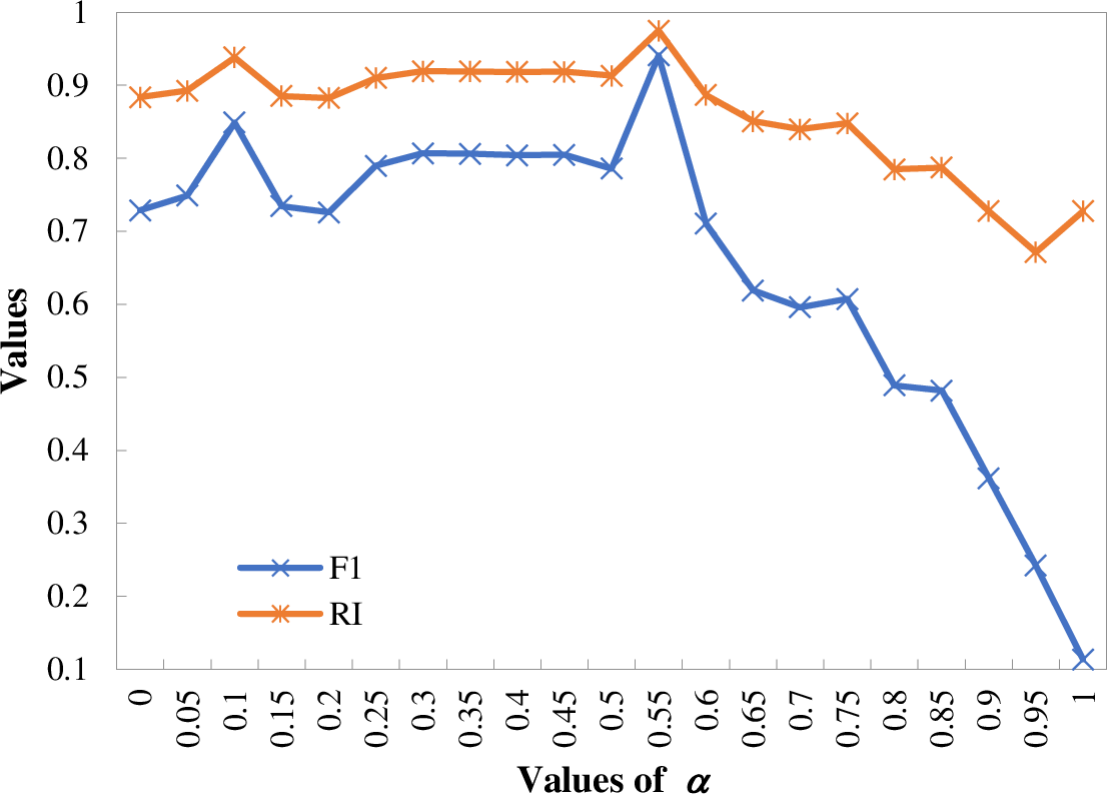
Values of F1 measure and RI with different values of parameter *α*.

Previous studies also analyzed the influence of different weights of citation- and text-based similarities in the hybrid similarities. Meng et al. [20] found that setting the same weights of two single similarities can obtain the best clustering results. Glänzel and Thijs [10,19] found that choosing the weights of citation-based similarity as 0.875 and 0.833 can obtain a balanced combination of two types of similarities. Meyer-Brötz et al. [30] found that decreasing the textual weight can obtain a more coherent clustering result and set the weights as 0.5 or 0.6 to obtain the best result. In this study, Fig 4 shows the F1 measure and RI values with different values of parameter *α*. It can be observed that both the highest F1 measure and RI values can be obtained when setting *α* = 0.55, which means that the weight of the citation-based similarity is slightly higher than that of the text-based similarity in the proposed model. Therefore, the proposed model in this study is further analyzed based on the optimal setting of parameters (*α* = 0.55, *β* = 0.45, and *λ* = 0.15) in the following sections.

### Comparison of different methods

To measure the performance of the proposed hybrid clustering model, four groups of comparisons of different clustering methods are analyzed. The first group is the comparison of the proposed hybrid clustering with the citation-based and text-based clustering methods to analyze the difference between hybrid clustering and “single clustering” methods (which means only considering citation-based similarity or text-based similarity). The second group is the comparison of different citation-based similarities in the hybrid clustering model to analyze the influence of different citation-based similarities. The third group is the comparison of different text-based similarities in the hybrid clustering model to analyze the influence of different text-based similarities, and the last group is the comparison of different hybrid clustering models, which had been studied in previous research and only considers textual statistical feature based on TF-IDF in the calculation of text-based similarity. The detailed settings of different clustering models are listed in Table 3.

**Table 3:** Different clustering methods with different parameter settings.

Groups	Methods	Parameter sets	Citation networks	Textual features
**1**	Amsler+TF-IDF+OA	*α* = 0.55, *β* = 0.45, *λ* = 0.15	Amsler	Statistical and topological features
	Amsler	*α* = 1, *β* = 0	Amsler	—
	TF-IDF+OA	*α* = 0, *β* = 1, *λ* = 0.15	—	Statistical and topological features
**2**	Amsler+TF-IDF+OA	*α* = 0.55, *β* = 0.45, *λ* = 0.15	Amsler	Statistical and topological features
	BC+TF-IDF+OA	*α* = 0.55, *β* = 0.45, *λ* = 0.15	Bibliographic coupling	Statistical and topological features
	CoC+TF-IDF+OA	*α* = 0.55, *β* = 0.45, *λ* = 0.15	Co-citation	Statistical and topological features
**3**	Amsler+TF-IDF+OA	*α* = 0.55, *β* = 0.45, *λ* = 0.15	Amsler	Statistical and topological features
	Amsler+TF-IDF	*α* = 0.55, *β* = 0.45, *λ* = 1	Amsler	Statistical feature
	Amsler+OA	*α* = 0.55, *β* = 0.45, *λ* = 0	Amsler	Topological feature
**4**	Amsler+TF-IDF+OA	*α* = 0.55, *β* = 0.45, *λ* = 1	Amsler	Statistical and topological features
	BC+TF-IDF	*α* = 0.55, *β* = 0.45, *λ* = 1	Bibliographic coupling	Statistical feature
	CoC+TF-IDF	*α* = 0.55, *β* = 0.45, *λ* = 1	Co-citation	Statistical feature

**Fig 5:**
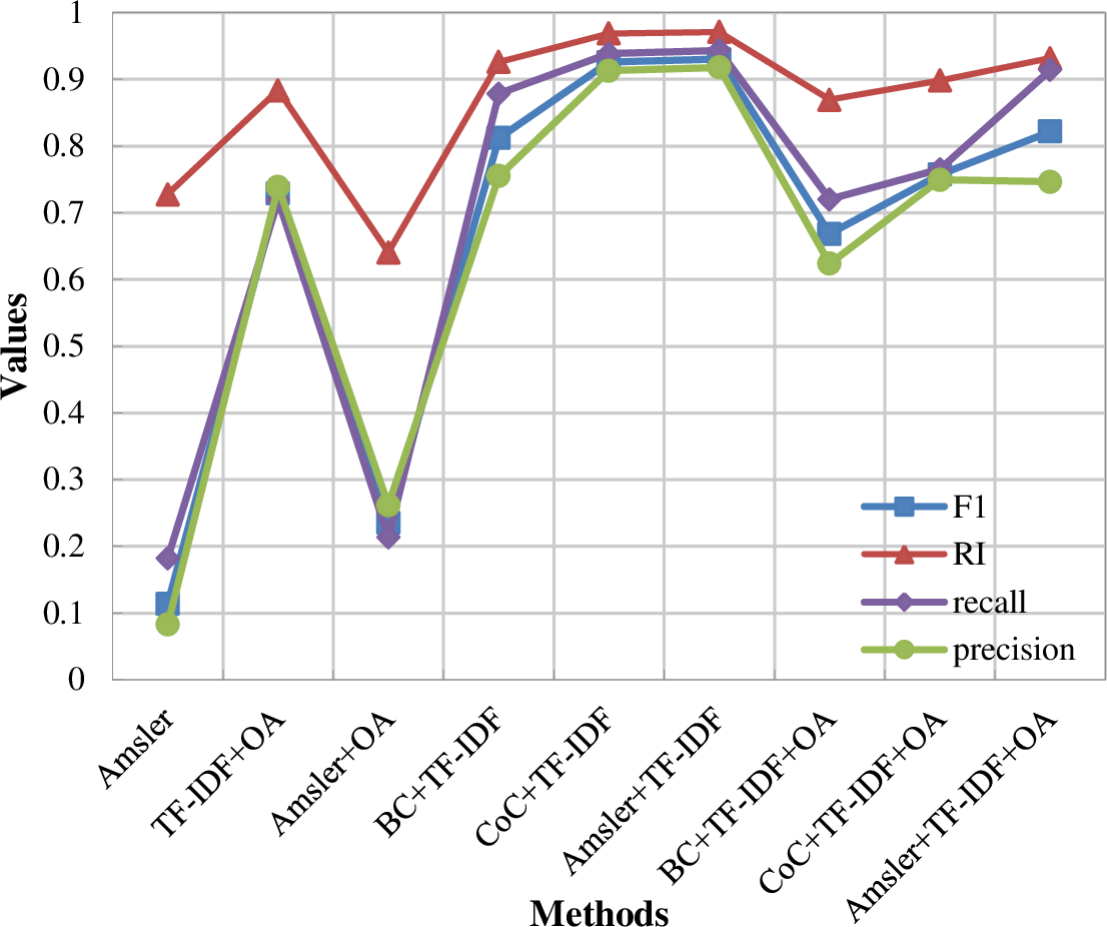
Values of evaluation measures of different methods. The evaluation measures are precision, recall, F1 measure, and RI values, and the different methods are the models listed in Table 3.

Fig 5 shows the values of evaluation measures of the different methods, i.e., precision, recall, F1 measure, and RI values, based on the benchmark. Overall, the curvilinear trends of these four metrics are similar, and the values of RI are higher than those of the other three metrics, respectively. The method that used only the citation-based similarity based on Amsler performs the worst. The second worst method is that which combines the citation-based similarity based on Amsler and the text-based similarity based on accessibility. The four groups with different parameter settings are analyzed in detail in the following.

#### Group #1: Comparison of hybrid clustering and single clustering methods

A comparison of the proposed hybrid clustering method (Amsler+TF-IDF+OA) with the single citation- (Amsler) and text-based (TF-IDF+OA) clustering methods shows that the values of all four metrics of the hybrid method are higher than those of the two single methods. This indicates that combining the citation- and text-based similarities can improve the clustering performance. It should be noted that the citation-based method (Amsler) has a very low precision, recall, and F1 measure, which means that the relation between the citation-based method based on the Amsler network and the benchmark considering the text-based similarity based on TF-IDF is weak; therefore, the difference between the citation-based and text-based clustering methods is highly significant. Similarly, the difference between the citation-based clustering method (Amsler) based on Amsler and the text-based clustering method (TF-IDF+OA) based on the hybrid textual feature is attributed to the weights of the TF-IDF, as well as the accessibility.

#### Group #2: Comparison of different citation-based similarities in hybrid clustering methods

The same text-based similarity consists of textual statistical and topological features and different citation-based similarities based on different citation networks, namely bibliographic coupling, co-citation, and Amsler. Different hybrid clustering methods with different citation-based similarities (BC+TF-IDF+OA, CoC+TF-IDF+OA, and Amsler+TF-IDF+OA) are compared based on this. As shown in Fig 5, the trends of the four metrics are approximately equivalent, of which the values of all four metrics of the Amsler+TF-IDF+OA method are the highest of the three hybrid clustering methods, and that of the BC+TF-IDF+OA method is the lowest. In other words, based on the aforementioned benchmark, the citation-based similarity that considers both the common out- and in-links between papers can obtain more relevant and coherent clustering results, and the performance of the hybrid clustering method based on the common in-links is considerably better than that of the hybrid method based on the common out-links between papers.

#### Group #3: Comparison of different text-based similarities in hybrid clustering methods

Similar to the second group, based on the same citation-based similarity and different text-based similarity based on different textual features, the hybrid clustering methods with different text-based similarities are compared. It is apparent from Fig 5 that the Amsler+OA method that only considers the textual topological feature in text-based similarity performs the worst, whereas the Amsler+TF-IDF method that only considers the textual statistical feature in text-based similarity performs the best. It means that the text-based similarity with both the textual statistical and topological features performs worse than that which only considers the textual statistical feature in the hybrid clustering methods based on the benchmark. The reason may be that the benchmark we built only considers the text-based similarity based on the textual statistical feature. Considering that the performance of the proposed method remains far better and more reasonable, the proposed model is still effective.

#### Group #4: Comparison of different hybrid clustering methods

In this group, the two other hybrid clustering models are compared with the proposed hybrid clustering model in this study. The BC+TF-IDF model clusters documents according to the hybrid similarities consisting of the citation-based similarity based on bibliographic coupling links and the text-based similarity based on the cosine measure of TF-IDF, i.e., the concept of the “core documents” proposed by Glänzel and Thijs [10]. The CoC+TF-IDF model is similar to the preceding model; the difference is that the citation-based similarity is based on the co-citation links between papers that have been analyzed and its performance was found to be worse than the former by Glänzel and Thijs [10]. As shown in Fig 5, the values of the metrics of the CoC+TF-IDF model are the highest, i.e., the model based on co-citation and TF-IDF performs the best. It is apparent that the result obtained is the inverse of that by Glänzel and Thijs [10]. This may be attributed to the different distribution of networks based on different datasets, or the influence of the benchmark built in this study.

Generally, the hybrid clustering methods perform better than the other methods that only consider single citation- or text-based similarity. The hybrid clustering methods based on the Amsler network with both the common out- and in-links between papers perform much better than the hybrid methods based on networks that only involve out-links or in-links, respectively. Because the benchmark used in this study is set by the authors based on previous studies, the comparison between different clustering methods can only prove that the proposed hybrid clustering model is effective and can obtain reasonable clustering results, rather than demonstrating that it has the best clustering performance.

### Research topics in DEA field based on the proposed model

**Fig 6:**
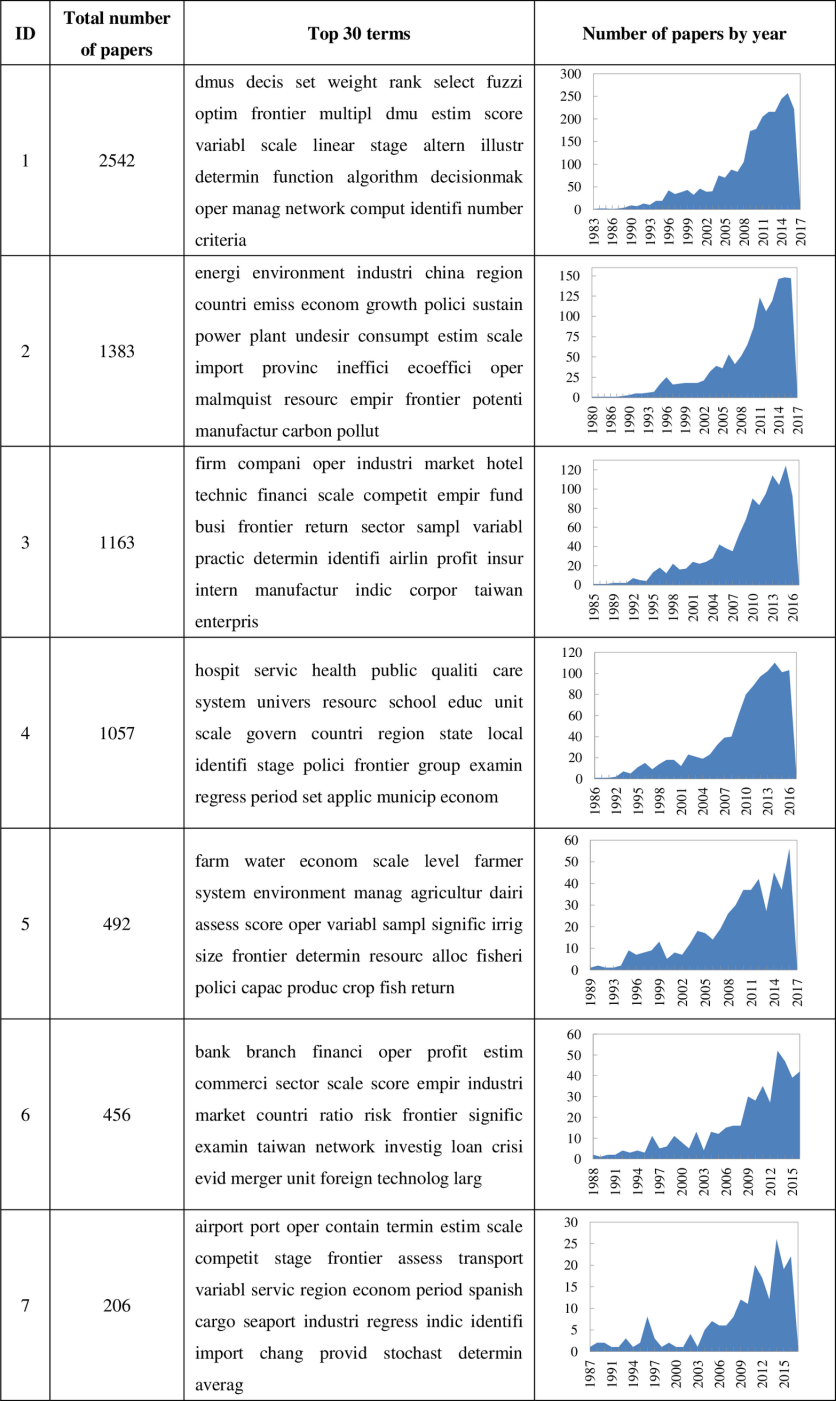
Information of each cluster based on the proposed model. It includes the total number of papers, top 30 high-frequency terms, and the trends of number of papers by year of the main seven clusters.

The clustering results using the proposed hybrid clustering model include seven main clusters, and exclude a small cluster, with only four papers based on the dataset used in this study. To further analyze the clustering result of the proposed model, i.e., research topics of the DEA field, the clusters are labeled according to the terms extracted from the title and abstract based on the term frequency. After filtering some common nouns and verbs in scientific articles, such as “study,” “paper,” “result,” “model,” and the common concepts related to DEA, such as “input,” “output,” “cost,” “efficiency,” “dea,” and “performance,” the top 30 high-frequency terms of each cluster were determined as shown in Fig 6. In addition, the number of papers per year for each cluster is also shown to analyze the development tendency of each research topic in the DEA field.

As shown in Fig 6, of the seven clusters, there are three clusters with less than 500 papers, three clusters with approximately 1000 papers, and only one cluster with more than 2000 papers. Based on the top 30 terms listed in Fig 6, the detailed research topics in the DEA field can be concluded as follows.

#### Cluster #1: Decision-making analysis and fuzzy DEA

According to the terms “dmus” (represents “decision-making units”) and “rank,” it can be initially concluded that this cluster includes the topic of ranking decision-making units using DEA. Based on these terms, including “decis,” “multipl,” “criteria,” “decisionmak,” and “oper,” multiple criteria decision-making is included in this research topic. The term “fuzzi” indicates that fuzzy DEA is also attracting attention, such that this cluster studies mainly the mutual application between decision making and DEA methods. Moreover, this cluster is the biggest cluster that indicates the trend where the number of papers is continually increasing, particularly after 2002.

#### Cluster #2: Energy and environment

This cluster focuses on studying the application of DEA in energy and environment according to the terms listed, such as “energi,” “environment,” “emiss,” “carbon,” “pollut,” and “plant.” In the energy and environment subfield, some undesirable factors such as carbon emission, pollution, and resource consumption are particularly of special concern. This research topic has been attracting attention since 1990, and has continued receiving increasing attention, particularly after 2008, according to the trend of a number of papers per year. In addition, the terms “China,” “region,” and “countri” indicate that this topic focuses most on studying the energy and environmental problems in specific countries or regions.

#### Cluster #3: Business company

This cluster is related to the application of DEA in a commercial company, i.e., a profitable organizations according to the terms “firm,” “company,” “market,” “financi,” “busi,” and “profit.” The detailed industries include hotel, airline, insurance, and manufacturing service. Applying the DEA methods to business areas can promote enterprises to obtain maximum profit and enhance the competitiveness of firms. The trend of number of papers per year is slightly fluctuant, and is growing more rapidly after 2009 based on the dataset.

#### Cluster #4: Public service

From the terms listed in Fig 6, such as “hospit,” “health,” “public,” “univers,” “school,” and “edu,” the research topic of this cluster is related to the application of DEA in the healthcare and education sectors, i.e., applying DEA methods to measure the efficiency of care services in hospitals and education quality in schools and universities. Because there are also some terms related to local government, such as “countri,” “region,” “state,” “local,” and “municip,” the research topic of this cluster also concerned other public affairs related to the local government. The trend of number of papers per year shows that it keeps increasing, and is growing rapidly after 2008.

#### Cluster #5: Agriculture and farm

Of the top 30 terms in this cluster, there are some terms closely related to agriculture and farm, such as “farm,” “water,” “farmer,” “agricultur,” “dairi,” “fisheri,” and “crop.” Therefore, the research topic of this cluster is the application of DEA in the agriculture and farm field. Applying DEA to the agriculture and farm fields can assess the efficiency of dairy farms and increase the efficiency of resource allocation. There are 492 papers in this cluster, which means that this research topic receives considerably less attention in the DEA field. The trend of the number of papers by year is slightly fluctuant, and it began to attract substantially more attention in 2008.

#### Cluster #6: Banking

According to the top term “bank,” this cluster can initially be judged to be related to the application of DEA in the banking industry. The terms, including “financi,” “commerci,” “loan,” and “crisi,” are also related to the banking industry. Applying the DEA methods to estimate the efficiency of branches of banks in countries around the world is also a popular topic in the application of DEA. The trend of number of papers per year is slightly fluctuant and reached its peak in 2013.

#### Cluster #7: Transportation

This cluster is the smallest of the seven clusters, which is related to the application of DEA in transportation based on the terms listed in Fig 6, such as “airport,” “port,” “transport,” “cargo,” and “seaport.” Applying DEA to the airport field can evaluate the performance of seaports, or airports, and the efficiency of airline industries. The trend of this research topic is also fluctuant; it has been studied since 1987, starting with only one paper per year, and now has increased to approximately 20 papers per year.

Overall, the clusters obtained using the proposed model are mostly related to the applications of DEA in different sectors, including energy and environment, agriculture and farm, transportation, banking, public service, and business companies. Of these application areas, the research topic related to the energy and environment sector is the biggest cluster. In addition to the application areas, there is also a topic related to the mutual application between decision making and DEA. Moreover, the listed terms related to the DEA methods include return to scale, stochastic frontier analysis, and Malmquist index.

## Discussion and conclusions

In this study, the authors propose a hybrid self-optimized clustering model that combines both citation links and textual features between papers to detect research topics in a specific research field. The proposed model has several improvements over the hybrid clustering models of previous studies. First, the Amsler network, which considers both bibliographic coupling and co-citation links, was constructed based on the citation links between papers. The citation links were used to compute the citation-based similarity between papers based on the cosine measure. Second, the textual feature was used to calculate the text-based similarity between papers, which considers both the textual statistical and topological features. Finally, the cosine angles underlying the linear combination of citation- and text-based similarity were considered as the hybrid similarity and used for further clustering using the Louvain method, which is based on modularity optimization.

To test the effectiveness of the proposed model, a case study related to the DEA field was analyzed in this study. Based on the dataset used, first, the optimal parameters related to the weights of citation- and text-based similarities were analyzed according to the F1 measure and RI metrics. Different parameter sets have significant influences on the clustering results, which were also analyzed by Meng et al. [20] and Meyer-Brötz et al. [30]. Based on the optimal weight of the citation-based similarity, i.e., setting 0.55 as the weight of citation-based similarity in the hybrid similarity, four comparative groups with different parameter sets were discussed. The evaluation measures (precision, recall, F1 measure, and RI) were analyzed to compare the different methods. The comparative results show that the different hybrid clustering methods based on different citation links or textual features have different clustering results, and the proposed model can also obtain reasonable and effective clustering results.

Admittedly, this study also has limitations. First, as there are no existing gold standards of clustering results based on the dataset we used, the proposed model can only be judged as an effective model, rather than being the best model based on the comparison results. According to Boyack et al. [49], the current challenge of topic identification aims to gain more information about the proposed clustering methods and the comparison to other methods, rather than obtaining the best clustering results of the dataset. This is because it is impossible to have only one single best solution. Therefore, based on the analysis and discussion in this study, the proposed hybrid clustering model still can provide inspiration for other related studies in the future. Second, we focus on extending and analyzing the computation of hybrid similarity between papers and then applying the Louvain method to cluster papers, whereas the comparison between different clustering algorithms is not included in this study, as the clustering algorithms also have influence on the performance of the clustering models. There are some new proposed clustering techniques for textual clustering, such as the aforementioned SSC methods, and fuzzy c-means methods [50,51]. In future works, first, we will aim to apply different clustering algorithms to cluster papers and analyze their advantages and disadvantages. This can help to build more efficient and effective clustering models. Second, different textual topological features and optimization objective functions will be introduced to the hybrid clustering methods to improve the performance of the clustering papers. Moreover, in our previous work [52], PageRank algorithm was applied to calculate the prestige of papers in the paper citation network that considering both the quantity and the quality of citations, similar idea will be used to calculate the citation-based similarity in the future.

## Supporting information

S1 Data Detailed statistics of DEA-related papers per year from 1980 to 2017. (XLSX)

S2 Data Detailed data of the F1 measure and RI with different values of parameter *α*. It also includes the values of *tp*, *fp*, *tn*, *fn*, precision, and recall.
(XLSX)

S3 Data Detailed data of evaluation measures of the different methods. The evaluation measures include precision, recall, F1 measure, and RI, and the different methods include the models listed in Table 3.
(XLSX)

S4 Data Detailed statistics of the number of papers by year of each cluster. (XLSX)

S5 Data Terms and its frequency of each cluster for labeling clusters. (XLS)

S6 Data Papers citation networks and terms. This zip file includes four text files, they are: 1) the citation number (CN) and reference number (RN) of all 7308 papers; 2) papers bibliographic coupling network; 3) papers co-citation network; 4) the terms extracted from the titles and abstracts of papers.
(ZIP)

S7 Data Basic information of papers. This data includes the ID, name, title, abstract, publication year, times cited, cited reference count, cited year, ISI unique article identifier, source, and subject category of 7308 papers related to DEA field.
(XLSX)
